# Interplay between *DMD* Point Mutations and Splicing Signals in Dystrophinopathy Phenotypes

**DOI:** 10.1371/journal.pone.0059916

**Published:** 2013-03-25

**Authors:** Jonàs Juan-Mateu, Lidia González-Quereda, Maria José Rodríguez, Edgard Verdura, Kira Lázaro, Cristina Jou, Andrés Nascimento, Cecilia Jiménez-Mallebrera, Jaume Colomer, Soledad Monges, Fabiana Lubieniecki, Maria Eugenia Foncuberta, Samuel Ignacio Pascual-Pascual, Jesús Molano, Montserrat Baiget, Pia Gallano

**Affiliations:** 1 Servei de Genètica, Hospital de la Santa Creu i Sant Pau and CIBERER U705, Barcelona, Spain; 2 Universitat de Barcelona (UB), Barcelona, Spain; 3 Servei d'Anatomia Patològica Hospital Sant Joan de Déu, Barcelona, Spain; 4 Unitat de Patologia Neuromuscular, Servei de Neurologia, Hospital Sant Joan de Déu, Barcelona, Spain; 5 Servicio de Neuropediatría, Hospital Nacional Pediátrico Garrahan, Buenos Aires, Argentina; 6 Servicio de Patología, Hospital Nacional Pediátrico Garrahan, Buenos Aires, Argentina; 7 Servicio de Genética, Hospital Nacional Pediátrico Garrahan, Buenos Aires, Argentina; 8 Servicio de Neurología Pediátrica, Hospital Universitario Materno Infantil La Paz, Madrid, Spain; 9 Unidad de Genética Molecular and CIBERER U753, Hospital Universitario Materno Infantil La Paz, Madrid, Spain; Johns Hopkins University School of Medicine, United States of America

## Abstract

*DMD* nonsense and frameshift mutations lead to severe Duchenne muscular dystrophy while in-frame mutations lead to milder Becker muscular dystrophy. Exceptions are found in 10% of cases and the production of alternatively spliced transcripts is considered a key modifier of disease severity. Several exonic mutations have been shown to induce exon-skipping, while splice site mutations result in exon-skipping or activation of cryptic splice sites. However, factors determining the splicing pathway are still unclear. Point mutations provide valuable information regarding the regulation of pre-mRNA splicing and elements defining exon identity in the *DMD* gene. Here we provide a comprehensive analysis of 98 point mutations related to clinical phenotype and their effect on muscle mRNA and dystrophin expression. Aberrant splicing was found in 27 mutations due to alteration of splice sites or splicing regulatory elements. Bioinformatics analysis was performed to test the ability of the available algorithms to predict consequences on mRNA and to investigate the major factors that determine the splicing pathway in mutations affecting splicing signals. Our findings suggest that the splicing pathway is highly dependent on the interplay between splice site strength and density of regulatory elements.

## Introduction

Dystrophinopathies are the most frequent neuromuscular disorder. They are caused by mutations in the *DMD* gene, one of the largest genes found in humans [1], [2]. *DMD* encodes for dystrophin, a key player in the stabilization of the sarcolemma during muscle contraction [3]. Clinical phenotypes include severe Duchenne muscular dystrophy (DMD), milder Becker muscular dystrophy (BMD), intermediate muscular dystrophy (IMD) and pure cardiac X-linked dilated cardiomyopathy (XLCM). DMD is characterized by early-onset, rapidly progressive muscular weakness, leading to wheel-chair dependency before age 13 and death during the third decade. BMD is clinically heterogeneous but presents a later onset and slower progression [4].

Clinical severity is determined by the maintenance of the open reading-frame, allowing the expression of semi-functional dystrophin with preserved N-term and C-term protein-binding domains [5]. Some parts of the central rod-domain can be truncated with minimal impact on protein function [6]. Frameshift and nonsense mutations cause absence of dystrophin expression and a DMD phenotype. In-frame mutations lead to abnormal or reduced dystrophin in muscle causing BMD. On this particular feature is based the promising molecular therapy of antisense oligonucleotide (AON)–mediated exon-skipping. Targeting splicing motifs of the pre-mRNA can induce the exclusion of selected exons and restoration of an open reading-frame, theoretically allowing the conversion of DMD to the BMD phenotype [7]–[9].

Until recent years, molecular diagnosis was mainly limited to detection of exonic deletions and duplications accounting for 65–70% of all disease-causing mutations [10], [11]. Detection of the remaining 25–30% single point mutations or small rearrangements have historically been challenging due to the large size of *DMD* gene. Development of high-throughput screening methodologies has allowed routine diagnosis of these mutations [12]. However, the mutation impact on pre-mRNA splicing and protein expression is often unknown.

Exceptions to the reading-frame rule are found in approximately 9% of patients and the production of alternatively spliced transcripts is considered a key modifier of the clinical severity [10]. Skipping of the mutated exon has been reported in several nonsense BMD-associated mutations, suggesting a model based on disruption/creation of splicing regulatory elements (SRE) [13]–[18]. However, some findings suggest that SRE alteration is not the only factor determining exon-skipping [19]–[22]. Recently, Flanigan and co-workers postulated that exon-skipping occurs in a subset of weakly defined *DMD* exons [23]. It has also been found that splice site mutations can lead to exon-skipping or activation of cryptic splice sites [24]–[26]. Nevertheless, the main factors determining the final splicing pathway are still unclear.

The precise definition of *DMD* point mutations and their consequences help to improve our understanding of the molecular pathology in dystrophinopathies. Due to its particular features and size, *DMD* is a suitable model gene for the study of the in vivo effects of DNA variants on mRNA and the elements involved in the regulation of the splicing process. Point mutations also provide valuable information regarding critical protein domains for dystrophin function. Herein we report our results concerning the clinical phenotype, dystrophin expression and *DMD* molecular analysis in 105 dystrophinopathy patients, presenting 98 different point mutations. Muscle mRNA analysis performed in most patients, identified 27 mutations causing aberrant pre-mRNA splicing. The mechanisms involved in the development of splicing defects included abrogation of natural splice sites, creation of new splice sites, alteration of SREs and pseudoexon activation. Bioinformatics analysis using splice site and SRE predictive matrices was performed to investigate the major factors determining the splicing pathway in splice site mutations and the ability of available algorithms to predict exon-skipping events in exonic mutations.

## Materials and Methods

### Patient selection

Dystrophinopathy patients who tested negative for intragenic deletions and duplications were screened for point mutations using genomic DNA or muscle cDNA whole gene sequencing. Male patients were grouped into four phenotypic categories: DMD, BMD, IMD, and XLCM according to clinical presentation, family history, age at onset, progression and age at loss of ambulation (DMD<13, BMD≥16, IMD ≥13 and <16). Females expressing myopathic symptoms were reported as MC (manifesting carriers) while unaffected females were reported as AC (asymptomatic carriers). Patients or their parents in case of children gave written individual informed consent to participate in the study. The study was performed in accordance with the ethical standards laid down in the declaration of Helsinki and was approved by the Ethics Committee of Hospital de la Santa Creu i Sant Pau (HSCSP), Barcelona.

### Muscle biopsy analysis

A muscle biopsy was taken in 89 out of 105 cases. Muscle sections were analyzed using standard histological and immunohistochemical techniques, described elsewhere. Dystrophin IHC was performed using monoclonal antibodies against N-terminal (DYS3), rod-domain (DYS1) and C-terminal (DYS2) epitopes (Novocastra, Newcastle upon Tyne, UK). IHC analysis of other sarcolemmal proteins, such as α, β, γ and δ sarcoglycans, caveolin-3, dysferlin, utrophin and emerin, were also performed.

### Mutation detection

DNA was extracted from peripheral blood samples according to standard procedures. Prior to point mutation screening, DNA was tested for intragenic deletions and duplications by MLPA (multiple ligation-dependent probe amplification) (P034 and P035 Salsa Kit, MRC-Holland). Point mutation detection was performed on genomic DNA by direct sequencing of the 79 *DMD* exons and their flanking intronic sequences using SCAIP (single-condition amplification/internal primer) [12]. When muscle tissue was available, mutation analysis was first performed by cDNA sequencing and further confirmed on genomic DNA. Total mRNA was extracted and purified from approximately 30 mg of muscle using RNeasy Fibrous Tissue Mini Kit (Qiagen, Hilden, Germany) and subsequently retrotranscribed to cDNA by RT-PCR using polythymine primers (Invitrogen, Carlsbad, NM). Complete *DMD* cDNA was amplified and sequenced in twenty overlapping fragments using published [25] and self-designed primers. Sequencing analysis were performed using Big Dye 3.1 chemistry and ABI 3500×L equipment (Applied Biosystems, Foster City, CA). Nucleotide positions were determined according to the standard *DMD* reference sequence (GenBank accession number NM_004006.2), and mutation nomenclature follows the guidelines of the Human Genome Variation Society. In order to make data publicly available, mutations and associated phenotypic information were subtmited to the Leiden Open Variant Database (LOVD, www.dmd.nl), Leiden, the Neetherlands.

### Bioinformatics analysis

In silico analysis of wild-type and mutant sequences was performed using a variety of tools integrated in the Human Splicing Finder (HSF, http://www.umd.be/HSF/) [27] to identify potential splicing alterations. Acceptor (3′ ss) and donor (5′ ss) splice sites strength was scored using HSF [27], MaxEnt [28] and NNSPLICE [29] and SpliceSiteFinder (http://www.genet.sickkids.on.ca/ali/splicesitefinder.html) [30] matrices. These programs were used to predict disruption/creation of splice sites and identification of potential cryptic splice sites. Analysis SRE was done using different matrices that predict exonic splicing enhancer motifs (ESE), silencers motifs (ESS) or both: ESE-finder matrices for SR (serine/arginine-rich) protein binding sites [31], [32], Rescue-ESE hexamers [33], PESE and PESS octamers [34], EIE (exonic identity elements) and IIE (intronic identity elements) hexamers [35], Sironi's ESS motifs [36], Whang's ESS decamers and Fas-ESS hexamers [37], and Tra2b, 9G8, and hnRNP A1 binding site matrices [27]. These tools were used to predict mutation-associated SRE disruption/creation and to calculate the density of ESE and ESS motifs in wild-type *DMD* exons. The pathogenicity of amino acid substitutions was evaluated using four algorithms: Polyphen-2 [38], SIFT [39], Panther [40] and SNAP [41].

### Statistical analysis

The association between alteration of SRE motifs and milder BMD phenotype in truncating mutations was analyzed using Fisher's exact test for each SRE predictive matrix (Table S1). A permutation test was applied to significant SRE matrices to adjust for multiple comparisons. Differences in relative 5′ splice site strength and density of SRE motifs between exons exhibiting cryptic site activation and exon-skipping in 5′ ss mutations were analyzed using a paired T test.

### Analysis of splicing pathways

Mutations predicted to affect splice sites or SRE motifs were analyzed by semi-quantitative QF-PCR in muscle cDNA. For each mutation, specific fluorescent labelled primers pairs encompassing the mutated exon were designed (Table S2). PCR products were analyzed by capillary electrophoresis using ABI 3500×L equipment and Genemapper software (Applied Biosystems, Foster City, CA). Splicing outcomes were determined by comparing fragment length with position of potential cryptic splice sites and exon length. Peak area was used to calculate the relative ratio of each transcript population. Samples were run in duplicate together with a normal control.

## Results

**Figure 1:**
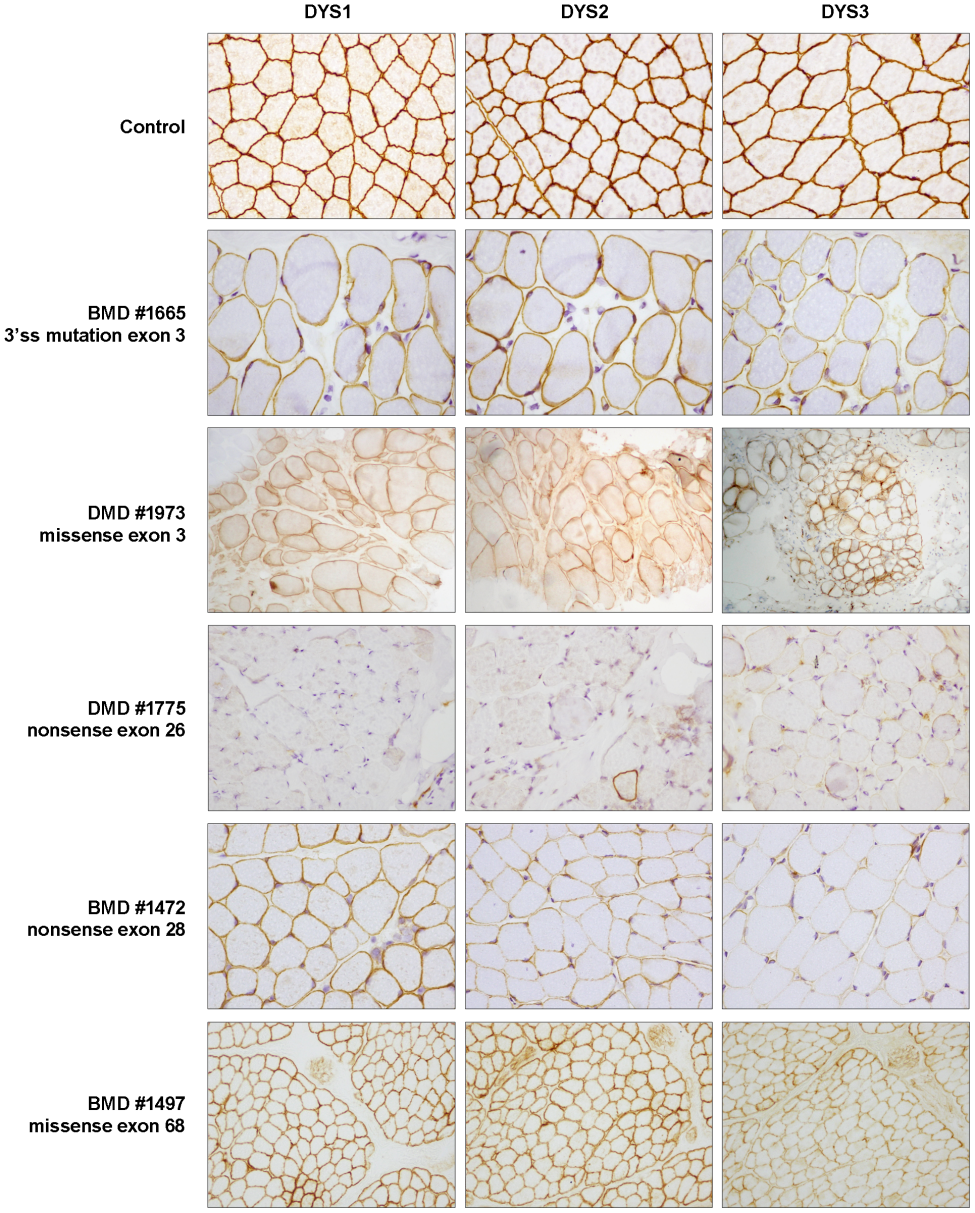
Representative results of dystrophin immunostaining. A healthy control and five patients presenting different types of *DMD* point mutations are shown. BMD patient #1665 shows dystrophin reduction. This patient presented a 3′ ss disrupting mutation causing mainly exon 3 in-frame skipping. Patient #1973 presents the rare combination of DMD phenotype and reduction of dystrophin expression. In this patient, a missense mutation in CH1 of ABD1 domain may cause impaired actin-binding activity. DMD patient #1775 carrying a nonsense mutation in exon 26 shows absence of dystrophin (an isolated revertant fibre can be observed in DYS2). In contrast, patient #1472 carrying a nonsense mutation in exon 28 shows reduced dystrophin expression and milder BMD phenotype. mRNA analysis in this patient revealed in-frame exon-skipping due to the disruption of an ESE motif. In the last row, BMD patient #1497 shows a very mild reduction of dystrophin expression. This patient presented a missense mutation in the ZZ domain that may compromise β-distroglycan binding.

We identified 98 different point mutations in 105 unrelated dystrophinopathy patients, 99 males and 6 female carriers. Table 1 shows the identified mutations and associated muscular phenotypes together with results regarding muscle dystrophin immunostaining and mRNA analysis. Representative images of dystrophin immunostaining are shown in Figure 1. We identified 54 nonsense mutations, 15 small deletions, 11 insertion/duplications, 20 splice site mutations, 4 missense mutations, and one deep intronic mutation. Aberrant splicing was found in 27 mutations through muscle cDNA and/or in silico analyses. Mechanisms involved in splicing defects included abrogation of 17 natural splice sites, two splice site creations, seven SRE alterations and one pseudoexon activation. In the male patients, 75 had DMD, 15 BMD, 8 IMD and one XLCM. In the female carriers, one was asymptomatic (AC) while five manifested myopathic symptoms (MC). The manifesting carriers reported here were included in a previous work concerning clinical outcomes and X-chromosome inactivation [42].

**Table 1:** Summary of *DMD* point mutations, clinical phenotype and muscle dystrophin immunostaining in 105 dystrophinopathy patients.

Patient	Phenotypic group	Dystrophin immunolabelling	Mutation at genomic DNA level	RT-PCR Fragments	Mutation at protein level	Exon	Protein Domain	Mutation frameness	Exon frameness
**Nonsense mutations**									
664	DMD	Absence	c.433C>T	r.433c>u	p.Arg145X	6	ABD: CH2	out	out
1279	DMD	Decreased intensity of DYS1 and DYS2, absence of DYS3	c.583C>T	Not performed	p.Arg195X	7	ABD: CH2	out	out
918	DMD	Absence	c.583C>T	r.583c>u	p.Arg195X	7	ABD: CH2	out	out
1614	MC	Absence with 1–15% of positive fibres	[c.724 C>T] + [ = ]	r.724c>u	p.Gln242X	8	ABD: CH2	out	out
468	BMD	Not performed	c.883C>T	Not performed	p.Arg295X	9	H1	out	in
1960	DMD	Absence	c.1388G>A	r.1388g>a	p.Trp463X	12	R2	out	out
1953	DMD	Absence	c.1474C>T	r.1474c>u	p.Gln492X	12	R2	out	out
1520	DMD	Absence	*c.1510C>T	r.1510c>u	p.Gln504X	13	R2	out	in
1957	DMD	Absence with isolated revertant fibres	*c.1638G>A	r.1638g>a	p.Trp546X	14	R2	out	in
1252	DMD	Absence	*c.2032C>T	r.2032c>u	p.Gln678X	17	H2	out	out
1774	DMD	Absence	*c.2215G>T	r.2215g>u	p.Glu739X	18	R4	out	out
820	DMD	Absence	c.2227C>T	r.2227c>u	p.Gln743X	18	R4	out	out
1490	DMD	Absence of DYS1, decreased intensity of DYS2 and DYS 3	*c.2518C>T	r.2518c>u	p.Gln840X	20	R5	out	out
1986	DMD	Absence with 3% of revertant fibres	*c.2560A>T	r.2560a>u	p.Lys854X	20	R5	out	out
1435	DMD	Absence	c.3427C>T	r.3427c>u	p.Gln1143X	25	R7	out	in
1900	DMD	Absence with <1% revertant fibres	*c.3511G>T	r.3511g>u	p.Glu1171X	26	R8	out	in
1775	DMD	Absence with isolated revertant fibres	*c.3578T>A	r.3578u>a	p.Leu1193X	26	R8	out	in
1643	AC	Isolated negative fibres	[c.3622C>T] + [ = ]	r.[ = , 3622c>u]	p.Gln1208X	27	R8	out	in
1472	BMD	Decreased intesity	*c.3850G>T	r.[3850g>t; 3787_3921del; 3787_4071del]	p.[Glu1284X; Glu1263_Asp1307del; Glu1263_Glu1357del]	28	R9	in/out	in
1491	IMD	Absence of DYS1, decreased intensity of DYS2 and DYS3	c.3982C>T	r.[3982c>u; 3922_4071; 3787_4071del]	p.[Gln1328X; Glu1263_Asp1307del; Glu1263_Glu1357del]	29	R9	in/out	in
1967	DMD	Absence with isolated revertant fibres	c.4099C>T	r.4099c>u	p.Gln1367X	30	R9	out	in
1511	DMD	Absence	c.4527T>G	r.4527u>g	p.Tyr1509X	33	R11	out	in
128	DMD	Not performed	*c.4558G>T	Not performed	p.Glu1520X	33	R11	out	in
1396	DMD	Abesence	*c.4838G>A	r.4838g>a	p.Trp1632X	34	R12	out	in
1580	DMD	Absence of DYS3, pronounced decreased intensity with negative fibres with DYS2 and DYS3	c.5131C>T	r.5131c>u	p.Gln1711X	36	R13	out	in
1958	DMD	Absence with isolated revertant fibres	c.5131C>T	r.5131c>u	p.Gln1711X	36	R13	out	in
1976	DMD	Absence with isolated revertant fibres	*c.5308A>T	r.5308a>u	p.Arg1770X	37	R13	out	in
548	BMD	Decreased intensity with isolated negative fibres	c.5287C>T	r.[5287c>u; 5155_5325del]	p.[Arg1763X ; Arg1719_Lys1775del]	37	R13	in/out	in
1728	BMD	Decreased intensity in mosaic pattern	c.5287C>T	r.[5287c>u; 5155_5325del]	p.[Arg1763X ; Arg1719_Lys1775del]	37	R13	in/out	in
1097	IMD	Not performed	c.5371C>T	Not performed	p.Gln1791X	38	R14	out	in
1624	DMD	Absence	c.5530C>T	r.5530c>u	p.Arg1844X	39	R14	out	in
1691	DMD	Absence	c.5530C>T	r.5530c>u	p.Arg1844X	39	R14	out	in
975	DMD	Absence	*c.5611A>T	r.5611a>u	p.Lys1871X	40	R14	out	in
374	DMD	Absence	c.5646C>A	r.5646c>a	p.Tyr1882X	40	R15	out	in
1221	MC	Isolated negative and decreased intensity fibres	[ = ] ; [c.5893C>T]	r.[ = ; 5893c>u]	p.[ = ; Gln1965X]	41	R15	out	in
444	DMD	Not performed	c.6283C>T	Not performed	p.Arg2095X	43	R16	out	out
1461	XL-CM	Mosaic pattern with 8% negative fibres	c.[ = ; 6292C>T]	r.[ = ;6292c>u]	p.[ = ;Arg2098X]	44	R16	out	out
1743	DMD	Absence with isolated revertant fibres	c.6292C>T	Not performed	p.Arg2098X	44	R16	out	out
1837	DMD	Not performed	*c.6352C>T	Not performed	p.Gln2118X	44	R17	out	out
1970	DMD	Absence	c.6973C>T	r.6973c>u	p.Gln2325X	48	R19	out	in
1954	DMD	Absence	c.7564C>T	Not performed	p.Gln2522X	52	R20	out	out
1457	DMD	Absence	c.7657C>T	r.7657c>u	p.Arg2553X	52	R20	out	out
101	DMD	Not performed	c.7657C>T	Not performed	p.Arg2553X	52	R20	out	out
466	DMD	Absence	c.8608C>T	r.8608c>u	p.Arg2870X	58	R23	out	out
602	DMD	Absence	c.8713C>T	r.8713c>u	p.Arg2905X	59	R23	out	out
1256	DMD	Absence	c.8944C>T	r.8944c>u	p.Arg2982X	60	R24	out	in
1955	DMD	Absence	c.9100C>T	r.9100c>u	p.Arg3034X	61	R24	out	out
1523	DMD	Absence	c.9148C>T	r.9148c>u	p.Gln3050X	61	H4	out	out
1175	DMD	Not performed	c.9337C>T	Not performed	p.Arg3113X	64	CRD	out	in
1344	DMD	Absence of DYS2 and DYS3. Decreased intensity of DYS1	c.9380C>G	Not performed	p.Ser3127X	65	CRD	out	out
1754	DMD	Not performed	*c.9542G>A	Not performed	p.Trp3181X	65	CRD: EF-2	out	out
996	DMD	Absence	c.9568C>T	r.9568c>u	p.Arg3190X	66	CRD: EF-2	out	out
1956	DMD	Absence	c.10033C>T	r.10033c>u	p.Arg3345X	69	CRD: ZZ	out	out
1965	DMD	Absence	*c.10147A>T	r.10147a>u	p.Lys3383x	70	CTD	out	out
**Deletions mutations**									
1968	DMD	Absence	*c.39del	Not performed	p.Glu14LysfsX12	2	ABD: CH1	out	out
1839	DMD	Absence	*c.114_115del	r.114_115del	p.Asn39ProfsX4	3	ABD: CH1	out	in
1558	DMD	Pronounced decreased intensity	c.174_175del	r.174_175del	p.Gly59Ala_fsX29	3	ABD: CH1	out	in
1556	DMD	Absence	*c.5613del	not performed	p.Ala1872LeufsX2	40	R14	out	in
1966	DMD	Absence	*c.6127del	r.6127del	p.Asp2043IlefsX30	43	R16	out	out
1734	DMD	not performed	c.6128_6131del	not performed	p.Asp2043ValfsX29	43	R16	out	out
1729	DMD	Absence with 4% of revertant fibres	*c.6580_6614del	r.6580_6614del	p.Glu2194Ala_fsX17	45	R17	out	out
1314	MC	Isolated negative fibres	*[c.6638del] + [ = ]	r.[ = , 6638del]	p.Leu2213CysfsX8	46	R18	out	out
1963	DMD	Absence	*c.8034_8037del	r.8034_8037del	p.Glu2681LeufsX44	55	R21	out	out
1974	DMD	Absence	*c.9862del	r.9862del	p.Glu3288AsnfsX42	68	CRD	out	out
1547	DMD	Absence	*c.9885del	r.9885del	p.Val3297SerfsX33	68	CRD	out	out
1972	DMD	End-stage muscular dystrophy, dystrophin IHC not evaluable	c.10101_10103del	r.10101_10103del	p.Glu3367del	70	CTD	in	out
1242	BMD	Not performed	*c.10231_10235del	Not performed	p.Thr3411AspfsX20	71	CTD	out	in
605	BMD	Absence of DYS2, decreased intensity of DYS1 and DYS3	*c.10235del	r.[10235del; 10224_10262del]	p.[Leu3412ArgfsX7; Pro3409_Ala3421del]	71	CTD	in/out	in
1961	DMD	Absence	*c.10624_10625del	r.10624_10625del	p.Pro3542SerfsX2	75	CTD	out	out
**Duplications, insertions and delins mutations**									
1990	DMD	Not performed	*c.1183_1186delins18 [Alu-like insertion]	Not performed	p.Arg395CysfsX17	11	R1	out	out
1862	DMD	Not performed	*c.1510dup	Not performed	p.Gln504ProfsX15	13	R2	out	in
22	MC	End-stage muscular dystrophy, dystrophin IHC not avaluable	*c.2095delinsTC	Not performed	p.Ala699SerfsX21	17	H2	out	out
1741	DMD	Not performed	*c.2667dup	Not performed	p.Leu890IlefsX30	21	R5	out	out
1521	DMD	Absence	*c.5139_c.5140delinsT	r.5139_c.5140delag_insu	p.Lys1713AsnfsX8	36	R13	out	in
1632	IMD	Absence with isolated revertant fibres	*c.7360dup	r.7360dup	p.Thr2454AsnfsX37	51	H3	out	out
1735	DMD	Not performed	*c.8711_8715delinsAGG	Not performed	p.Leu2904GlnfsX5	59	R23	out	out
1148	DMD	Absence	*c.8955dup	Not performed	p.Ala2986CysfsX12	60	R24	out	in
1646	DMD	Not performed	*c.9348dup	Not performed	p.Lys3117GlufsX15	64	CRD	out	in
1964	DMD	Absence with isolated revertand fibres	*c.9583_9584insAT	r.9583_9584insat	p.Arg3195HisfsX89	66	CRD: EF-2	out	out
1736	BMD	Decreased intensity with DYS1 and DYS2. Absence of DYS3 with isolated positive fibres.	*c.10409dup	Not performed	p.Leu3470PhefsX21	74	CTD: SBS	out	in
**Splice site mutations**									
1665	BMD	Decreased intensity	c.94-1G>T	r.[94_105del; 94_186del]	p.[Phe32_Gln35del; Phe32_Leu62del]	3	ABD: CH1	in	in
2042	IMD	Decreased intensity	*c.265-1G>A	r.[265del; 265_367del]	p.[Val89LeufsX3; Val89_Gln119del]	5	ABD: CH1	in/out	in
1849	DMD	Absence	*c.358-1G>C	Not performed	p.spl	6	ABD: CH2	?	out
1753	IMD	Absence with isolated revertand fibres	*c.961-1G>A	Not performed	p.spl	10	R1	?	in
1971	DMD	Absence	c.1332-9A>G	r.[ = ; 1331_1332ins1332-8_1332-1; 1332_1359del; 1332_1482del; 1332_1602]	p.[ = ; Asn444LysfsX9; Asn444LysfsX5; p.Asn444LysfsX7]	12	R1-2	in/out	out
1982	DMD	Absence	c.1332-9A>G	r.[ = ; 1331_1332ins1332-8_1332-1; 1332_1359del; 1332_1482del; 1332_1602]	p.[ = ; Asn444LysfsX9; Asn444LysfsX5; p.Asn444LysfsX7]	12	R1-2	in/out	out
338	BMD	Decreased intensity	*c.1704+2T>A	r.[ = ; 1704_1705ins1704+1_1704+11; 1704_1705ins1704+1_1705-1]	p.[ = ; Cys569ValfsX18; Cys569ValfsX5]	14	R3	in/out	in
1746	DMD	Absence	*c.2169-2A>G	Not performed	p.spl	18	R4	?	out
1959	DMD	Absence	*c.2803+1del	r.[2623_2803del; 2757_2803del]	p.[Asp875PhefsX14; Lys919AsnfsX3]	21	R5-6	out	out
1342	BMD	Decreased intensity	*c.3432+3A>T	r.3277_r.3432del	p.Leu1093_Gln1144del	25	R7	in	in
642	DMD	Absence	*c.3603+1G>A	r.3603_3604ins3603+1_3603+116{3603+1G>A}	p.Arg1202ValfsX25	26	R8	out	in
1061	BMD	Absence of DYS2. Near normal intensity of DYS1 and DYS3.	c.3603+2dupT	r[ = ; 3603_3604ins3603+1_3603+116{3603+1G>A}]	p.[ = ; Arg1202ValfsX25]	26	R8	in/out	in
520	BMD	Decreased intensity with isolated negative fibres	*c.3786+1G>A	r.[3604_3786del; 3604_4071del]	p.[Arg1202_Glu1262del; Arg1202_Glu1357del]	27	R8	in	in
1915	IMD	Decreased intensity	c.4845+1G>A	Not performed	p.spl	34	R12	?	in
1339	DMD	Absence	*c.5444A>G	r.5444_r.5448del	p.Asp1815GlufsX2	38	R14	out	in
1962	DMD	Absence with isolated revertand fibres	*c.6614+1G>T	Not performed	p.spl	45	R17	?	out
632	MC	Mosaic pattern with 20% of negative fibres	[c.6913-1G>A] + [ = ]	r.[ = , 6913del]	p.[ = ; Val2305PhefsX16]	48	R18	out	in
1606	DMD	Absence	c.9563+1G>A	Not performed	p.spl	65	CRD: EF-2	?	out
1619	BMD	Decreased intensity with negative fibres	*c.9563+5G>C	r.[ = ; 9560_9563del; 9563_9564ins9563+1_9563+9]	p.[ = ; Asp3187GlyfsX95; Thr3188_Gly3189insTyrValTrp]	65	CRD: EF-2	in/out	out
1455	DMD	Absence	c.10086+5G>C	r.[ = ; 9975_10086del; 9808_10086del]	p.[ = ; Tyr3326LeufsX14; Ala3270_Pro3362del]	69	CRD: ZZ	in/out	out
**Deep Intronic mutations**									
1465	BMD	Decreased intensity	c.9225-647A>G	r.[ = ; 9224_9225ins9225-713_9225-647]	p.[ = ; Asn3075LysfsX3]	i62	CRD: WW	in/out	n.a.
**Missense mutations**									
1973	DMD	Decreased intensity with normal and negative isolated fibres	*c.158T>G	r.158u>g	p.Leu53Arg	3	ABD: CH1	in	in
1755	IMD	Not performed	*c.497G>T	Not performed	p.Gly166Val	6	ABD: CH2	in	out
1215	IMD	Decreased intensity with negative fibres	*c.1350_1351delGGinsAT	r.1350_1351delgginsat	p.Met450Ile_Asp451Tyr	12	R1	in	out
1497	BMD	Mildly decreased intensity of DYS3, DYS1 and DYS2 near normal	*c.9958C>T	r.9958c>u	p.Pro3320Ser	68	CRD: ZZ	in	out

Asterisks indicate novel mutations not previously reported in the LOVD (www.dmd.nl). Protein domains: ABD, actin-binding domain; CH1-2, calponin homology; R1-24, spectrin-like repeats; H1-4, hinge regions; CRD, cysteine-rich domain; WW, domain with a signature of two tryptophan that binds to proline-rich proteins; EF-1/2, EF-hand domains found in calcium-binding proteins; ZZ, zinc-finger domain; CTD, c-terminal domain; SBS α1-syntrophin-binding site.

The frequency of each type of mutation differed substantially between clinical phenotypes. Most DMD patients presented nonsense and frameshift truncating mutations that accounted for 84% of cases (63/75). Splice site mutations were found in 14.6% of cases (11/75) while missense and in-frame changes were found in 2.7% (2/75). BMD patients presented the same proportion of truncating mutations than splicing defects, 46.7% (7/15) each. Splicing defects in BMD include a pseudoexon activation caused by a deep intronic substitution. Only one missense mutation was detected in BMD patients (6.7%). IMD patients presented three truncating mutations (37.5%), three splice site mutations (37.5%) and two missense mutations (25%).

### Nonsense and frameshift mutations

**Figure 2:**
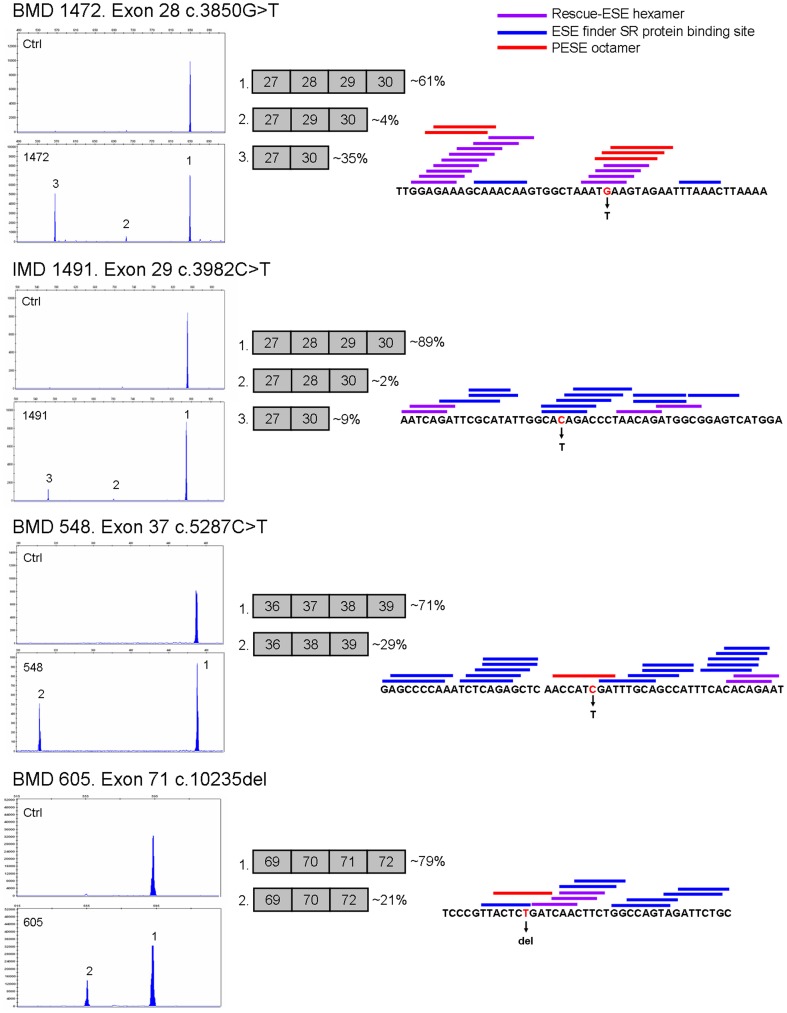
Exonic mutations associated with exon-skipping events. On the left, semi-quantification of alternative transcripts by QF-PCR on muscle biopsy cDNA. In the centre, schematic representation of the detected transcript species and their relative ratio. On the right, mutation sequence context and predicted ESE motifs: blue bars indicate ESE finder SR protein binding sites; violet bars indicate Rescue-ESE hexamers; red bars indicate PESE octamers. The mutated nucleotide is indicated in red.

The great majority of truncating mutations identified in male patients were associated with more severe DMD and IMD phenotypes (66/74, 89.2%). However, 8 patients exhibiting BMD or XLCM phenotypes presented nonsense or frameshift mutations (11%). Patient #444, presenting isolated DCM but no muscle weakness, carried a nonsense mutation in somatic mosaicism. BMD patients #468, #1472, #548, #1728, #1242, #605 and #1736 presented six different mutations localized in five in-frame skippable exons (exons 9, 28, 37, 71 and 74). Three of these mutations were found to induce significant amounts of in-frame exon-skipping in muscle cDNA (Figure 2). Patient #1472 presenting c.3850G>T in exon 28, showed ∼4% of exon 28 skipping and ∼35% of exons 28 and 29 double skipping. Patients #548 and #1728 carrying c.5287C>T in exon 37, showed ∼29% of exon 37 skipping. Patient #605 presenting c.10235del in exon 71, showed ∼21% of exon 71 skipping. In addition, in-frame exon-skipping was also found in an IMD patient (#1491). This patient carrying c.3982C>T nonsense mutation in exon 29, showed ∼2% of exon 29 skipping and ∼9% of exon 28 and 29 double skipping.

In silico analysis of SRE motifs showed five ESE disruptions and one ESS creation in BMD mutations. Disruption of at least one PESE octamer occurred in mutations c.883C>T (exon 9), c.3850G>T (exon 28), c.5287C>T (exon 37), c.10235del (exon 71) and c.10231_10235del (exon 71). Mutation c.10409dup (exon 74) was predicted to create an ESS according to PESS and Fas-ESS matrices. Five BMD mutations were predicted to create an intronic identity element (IIE). IMD mutation c.3982C>T (exon 29) was predicted to disrupt three SR-protein binding sites and to create an ESS according to hnRNP A1 and Sironi's matrices (Table S1). To investigate the ability of available matrices to predict critical regions for exon recognition and, to assess the association of SRE alterations with exon-skipping in BMD patients, all truncating mutations located in in-frame exons were tested against different matrices (Table S1). In male patients, 32 nonsense/frameshift mutations were identified in-frame skippable exons but only six were associated with milder BMD phenotype (18.75%). Statistically significant differences between DMD/IMD and BMD mutation groups were found using PESE and IIE matrices. Other matrices did not show any significant difference. Predicted disruption of PESE octamers occurred in 5 out of 6 BMD mutations (83.3%) and in 6 out of 26 DMD/IMD mutations (23%) (Fisher's Exact Test, *P*-value 0.0112). Seven mutations associated with DMD/IMD were found in exons where exon skipping events have been previously described (exons 25, 29, 37, 38, and 40). None of them was predicted to disrupt any PESE octamer. Creation of IIE hexamers occurred in 5 of 6 BMD mutations (83.3%) and in 7 of 26 DMD/IMD mutations (26.9%) (Fisher's Exact Test, *P*-value 0.0185). A permutation test corroborated the significant result between the PESE and IIE matrices (truncated *P*-value product 0.00659).

### Splice site mutations

We identified twenty different splice site mutations in twenty-one unrelated patients. Splicing pathways were determined in fourteen mutations through muscle cDNA sequencing and QF-PCR analysis. Detected transcript species, relative ratio and splice site predictions are summarized in table 2. Most mutations involved canonical AG/GT nucleotides disrupting natural splice sites (13/20, 65%). Four involved non-canonical nucleotides (20%), of which one disrupted the splice site while the other three reduced its efficiency. Creation of a new splice site was found in four mutations. Two of them also disrupted a natural site (c.265-1G>A and c.6913-1G>A), while the other two (c.1332-9A>G and c.5444A>G) created a strong splice site more efficient than the natural site. An intronic single-base substitution far from a natural splice site (647 bp) provoked the activation of a cryptic 5′ ss causing the inclusion of a 67 bp pseudoexon into the mature mRNA. Mutations affecting natural 5′ ss were more frequent (11/21, 52.4%) than those affecting natural 3′ ss (6/21, 28.6%).

**Table 2:** Splicing pathways in splice site mutations.

Patient	Phenotype	Exon	Mutation	QF-PCR detected transcripts	Ratio	HSF score (% variation)	MaxEnt score (% variation)	Transcript frameness
1665	BMD	3	c.94-1G>T	Exon 3 skipping	71%	wt 89.2→60.3 (−32%)	wt 8.3→−0.3 (−103%)	in
				Cryptic 3′ ss activation causing 12 bp deletion	29%	79.2	0	in
2042	IMD	5	c.265-1G>A	Exon 5 skipping	64%	wt 93→64 (−31%)	wt 12.6→3.9 (−69%)	in
				New 3′ ss creation causing 1 bp deletion	36%	48.7→77.6 (+59%)	−2.3→5.7 (+350%)	out
1971/1982	DMD	12	c.1332-9A>G	New 3′ ss creation causing 8 bp inclusion	58%	58.5→87.4 (+50%)	−6.6→2.1 (+132%)	out
				Exon 12 skipping	27%	-	-	out
				Cryptic 3′ ss activation causing 28 bp deletion	6%	83.5	2.9	out
				Normal splicing	5%	wt 83.1→83.2 (+0.14%)	wt 6.9→1 (−84%)	in
				Exon 12 and 13 skipping	4%	-	-	out
338	BMD	14	c.1704+2T>A	Cryptic GT 5′ ss activation causing 11 bp inclusion	60%	78.8	0.5	out
				Usage of exon 15 natural 5′ ss causing intron 14 retention	28%	wt 73.7	wt 6.4	out
				Normal splicing	12%	wt 90→63.2 (−30%)	wt 7→−1.2 (−117%)	in
1959	DMD	21	c.2803+1del	Exon 21 skipping	88%	wt 89.4→12.8 (−86%)	wt 10.1→−20 (−299%)	out
				Cryptic GC 5′ ss activation causing 47 bp deletion	12%	0	0	out
1342	BMD	25	c.3432+3A>T	Exon 25 skipping	100%	wt 77.1→72.1 (−7%)	wt 8.7→3.1 (−64%)	in
642	DMD	26	c.3603+1G>A	Cryptic GT 5′ ss activation causing 116 bp inclusion	100%	wt 84.6→57.7 (−37%)	wt 8.4→0.2 (−98%)	out
						72.7	1.5	
1061	BMD	26	c.3603+2dupT	Normal splicing	83%	wt 84.6→79.5 (−6%)	wt 8.4→2.7 (−68%)	in
				Cryptic GT 5′ ss activation causing 116 bp inclusion	17%	72.7	1.5	out
520	BMD	27	c.3786+1G>A	Exon 27 skipping	80%	wt 82.6→55.8 (−32%)	wt 1→−7.2 (−801%)	in
				Exon 27, 28 and 29 skipping	20%	-	-	in
1339	DMD	38	c.5444A>G	New GT 5′ ss creation causing 5 bp	100%	61.5→88.3 (+44%)	1.1→9.3 (+757%)	out
632	MC	48	c.6913-1G>A	New 3′ ss creation causing 1 bp deletion	100%	wt 98.7→69.7 (−29%)	wt 10.1→1.3 (−87%)	out
						55.1→84.1 (+53%)	−1.54→6.41 (+516%)	
1619	BMD	65	c.9563+5G>C	Normal splicing	49%	wt 78.2→66.2 (−15%)	wt 6.8→1.3 (−81%)	in
				Deletion of 4 bp	26%	0	0	out
				Cryptic GC 5′ ss activation causing 9 bp inclusion	25%	0	0	in
1455	DMD	69	c.10086+5G>C	Exon 69 skipping	46%	-	-	out
				Exon 68 and 69 skipping	46%	-	-	in
				Normal splicing	8%	wt 95.3→83.2 (−13%)	wt 10.9→−41.4 (−41%)	in
1465	BMD	intron 62	c.9225-647A>G	Cryptic GT 5′ ss activation causing 67 bp pseudoexon inclusion	74%	56.9→85.9 (+51%)	4.4→8.8 (+97.3%)	out
				Normal splicing	26%	-	-	in

Natural (wt) and cryptic splice sites were scored using Human Splicing Finder (HSF) and Maximum Entropy Scan matrices (MaxEnt).

The splicing pathway differed from one mutation to another. In most cases (11/14) variable levels of more than one alternative transcript were detected. Splicing outcomes included exon-skipping, cryptic or new splice site activation, intron retention and pseudoexon inclusion. Six mutations induced exclusively or mainly exon-skipping, seven induced activation of alternative splice sites and one mutation induced predominantly normal splicing (c.3603+2dupT). In most cases, the clinical phenotype and expression of dystrophin correlated with the absence/presence of significant amounts of normal and/or in-frame mRNA transcripts. This correlation was not observed in two cases presenting significant amounts of in-frame transcripts and a severe phenotype (patients #2042 and #1455). In both cases, most abundant in-frame transcripts presented truncated protein-binding domains, actin-binding (ABD) or zinc-finger (ZZ) domains respectively. Loss of coding sequences may also have an impact on protein folding or stability. The mutations that abolished the function of natural sites presented a score reduction between 7% and 86% using HSF, and between 64% and 801% using MaxEnt (Table 2). The mutations that reduced the site efficiency presented a score reduction between 7% and 15% using HSF, and between 41% and 81% using MaxEnt. Most of the activated cryptic or new sites were predicted by HSF or MaxEnt matrices (Table 2). However, two transcripts presented activation of GC 5′ ss (#1959 and #1619) that were only predicted by the SpliceSiteFinder algorithm. Two transcripts did not correlate with any potential 5′ ss (patients #338 and #1619).

**Figure 3:**
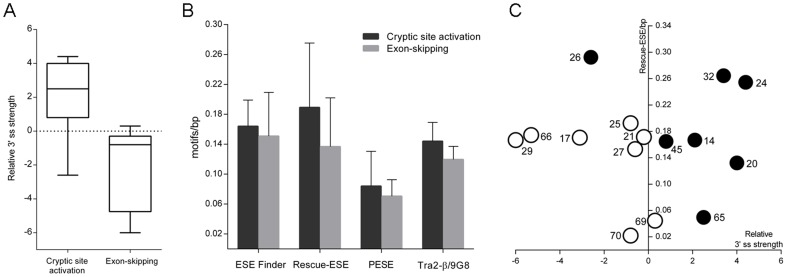
Factors determining the main splicing pathway in 5′ ss mutations: cryptic site activation versus exon-skipping. **A**) Relative 3′ ss strength (MaxEnt score difference with next distal natural 3′ ss) of exons exhibiting mainly cryptic site activation and exon exhibiting mainly exon-skipping. Box plots indicate the lowest and highest observation, lower and upper quartile, and median. **B**) Mean density of ESE motifs predicted by different matrices. **C**) Individual exons are plotted by the relative 3′ ss strength and density of Rescue-ESE motifs. Black-filled circles represent exons showing cryptic site activation. Non-filled circles represent exons showing exon-skipping. Exon numbers are indicated beside circles.

To investigate the major factors determining the main alternative splicing pathway in 5′ ss mutations (cryptic site activation versus exon-skipping), we analyzed several parameters: exon and intron length, density of SRE motifs, availability of cryptic sites and relative 3′ ss strength. The analysis was extended to eight exons with previously reported pathways in 5′ ss disrupting mutations [26]. We found that exons exhibiting mainly exon-skipping presented a weak 3′ ss, while most exons showing predominantly cryptic site activation presented a strong 3′ ss (Figure 3A, paired t test, *P*-value 0.0346). No statistically significant differences were found in other parameters. However, exons exhibiting cryptic site activation presented a mean density of ESE motifs higher than exon showing exon-skipping (Figure 3B).

### Missense and in-frame mutations

Four missense mutations and one amino acid deletion were detected in our cohort. All were located in highly conserved residues and were predicted to be pathogenic based on Polyphen-2 and SIFT algorithms. These mutations were not found in 100 healthy controls and not reported in the Exome Variant Server (http://evs.gs.washington.edu/EVS/). Two mutations were found in the N-terminal ABD. In the CH1 (calponin homology ABD domain), mutation p.Leu53Arg was found in a DMD patient with irregularly reduced expression of dystrophin (Figure 1). In the CH2 domain, mutation p.Gly166Val was found in a patient with IMD. In the central rod-domain at the spectrin-like repeat 2, a double amino acid change (p.Met450Ile_Asp451Tyr) was detected in an IMD patient presenting reduction of dystrophin with negative fibres. Two mutations were found in the C-terminal cysteine-rich region. Mutation p.Pro3320Ser in the ZZ-domain was found in a BMD patient with near normal dystrophin expression (Figure 1), and a single amino acid deletion, p.Glu3367del, was found in a DMD patient.

## Discussion

We describe a comprehensive analysis of 98 DMD point mutations related to clinical phenotype and their effect on muscle mRNA and dystrophin expression. Aberrant splicing was found in 27 mutations. Mechanisms responsible for the splicing defects consisted in abrogation of natural splice sites, creation of new splice sites, disruption/creation of regulatory elements (SRE) and pseudoexon activation. Bioinformatics analysis of nonsense/frameshift mutations revealed that PESE/PESS matrix is a powerful tool to predict critical regulatory regions for BMD-associated exon-skipping. Our findings suggest that the splicing pathway in 5′ ss disrupting mutations is highly dependent on the interplay between 3′ ss strength and density of exonic splicing enhancers.

In agreement with the reading frame rule, most nonsense and frameshift mutations in our cohort were found in patients presenting a severe DMD or IMD phenotype. However, 11% of them were detected in patients presenting milder BMD or XLCM phenotypes. Several mechanisms have been associated with the production of dystrophin in nonsense/frameshift mutations, ameliorating the clinical phenotype. These include alternative translation initiation in 5′ end mutations [43], [44], escape of nonsense-mediated mRNA decay (NMD) in mutations located in or beyond exon 74 [45] and somatic mosaicism [25], [46], [47]. However, the most reported mechanism is the skipping of the mutated exon, producing significant amounts of in-frame transcripts. Mechanisms involved in exon-skipping events include disruption and creation of SRE. Although creation of ESS has been reported [18], disruption of ESE is better documented [13]–[17], [25], [48]. Most widely used algorithms to predict ESE disruption are ESE Finder matrices for SR protein binding sites, and Rescue-ESE hexamers which are differentially present in exons and introns. However, the ability of these tools to predict exon-skipping events in the *DMD* gene is limited. Analysis of the mutation entries in the Leiden database (LOVD) revealed that ESE disruption occurred in 50% of BMD nonsense mutations [10]. Deburgrave et al. reported similar results, since 4 out of 8 mutations with confirmed mRNA exon-skipping had consequences on ESE motifs ESE motifs [25].

In our subset of patients, we found a nonsense mutation in somatic mosaicism in a patient who presented DCM but no muscle weakness. Clinical, pathological and molecular studies in this patient are discussed in greater detail in a previous work [49]. Exon-skipping events were found or predicted in seven BMD patients. We found that 8-mers putative splicing enhancers (PESE) and silencers (PESS) from Zhang and Chasin [34] are a powerful tool to predict in the *DMD* gene critical SRE motifs for exon recognition. PESE disruption was predicted in six patients presenting five different mutations. Disruption of ESE motifs was predicted only in one mutation when ESE finder or Rescue-ESE matrices were used (Figure 2 and Table S1). Surprisingly, disruption of PESE motifs in BMD overlapped in most cases with creation of an intronic identity element (IIE) [35], raising the possibility that the mutations had a double effect, contributing to loss of exon identity. An ESS creation was predicted by PESS and other matrices in a nonsense mutation in exon 74. However, in absence of cDNA studies we can not confirm an exon-skipping event, since mutations in this exon have been found to cause either exon-skipping or escape from NMD [14], [25]. None of the associated IMD/DMD mutations located in exons where exon-skipping events have previously been reported were predicted to disrupt any PESE octamer. However, one of these mutations located in exon 29 induced exon-skipping. Nevertheless, the proportion of exon-skipping transcripts in the patient was much lower than those found in a BMD patient presenting an identical skipping pattern (Figure 2), indicating that they are insufficient to rescue the phenotype. According to LOVD this mutation has been previously found in BMD patients, suggesting differences in the exon-skipping efficiency between individuals. In line with this hypothesis, Ginjaar et al. reported a BMD family with a nonsense mutation in exon 29 who presented variable phenotype severity, ranging from severe BMD to asymptomatic elevation of CK levels [17]. The authors reported that clinical variability was related to different levels of exon 29 skipping.

**Figure 4:**
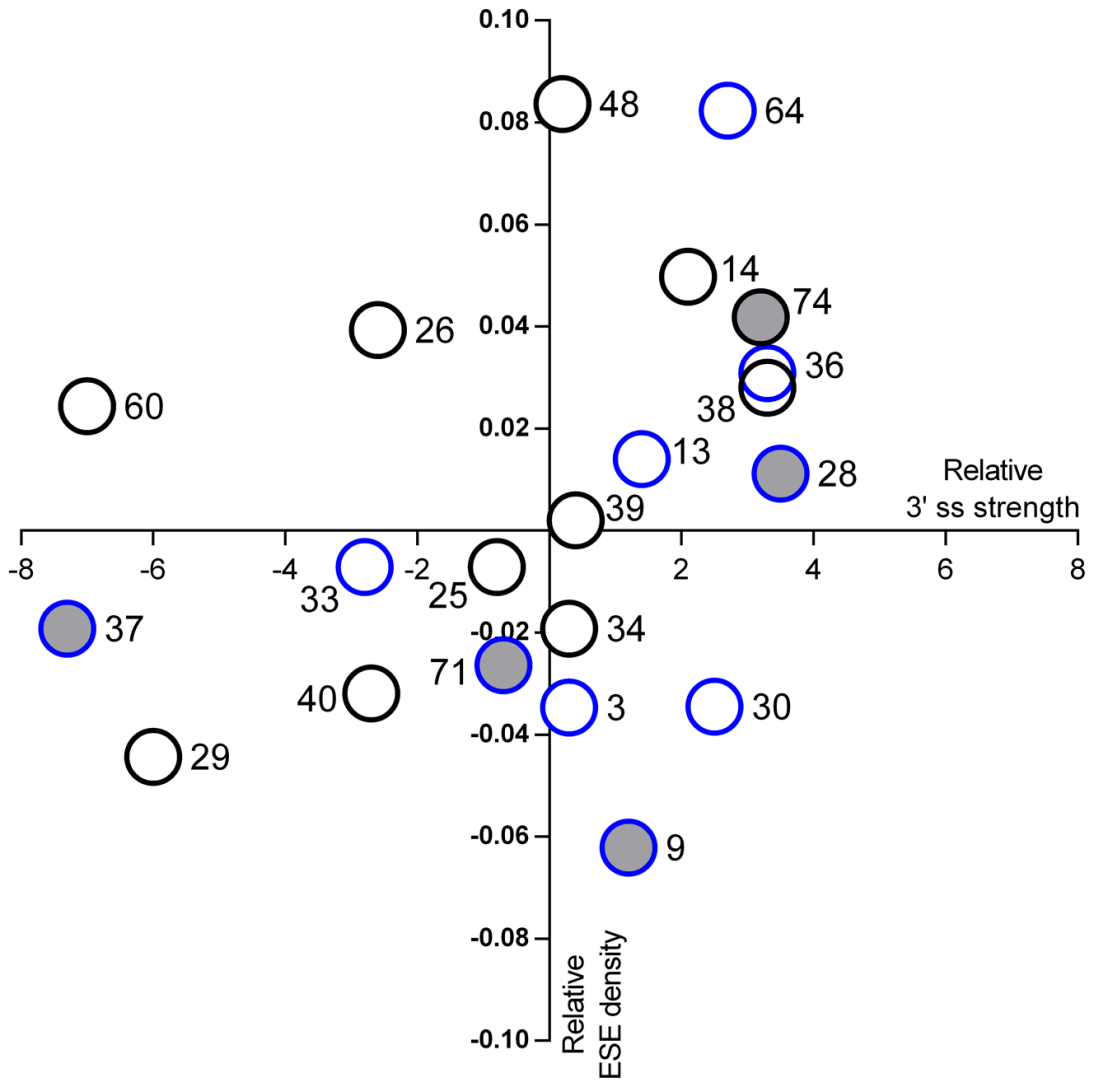
Relative ESE density versus relative 3′ ss strength in in-frame exons presenting nonsense and frameshift mutations. On the y axis: difference between exon PESE density and mean density of all *DMD* exons. On the x axis: difference between 3′ ss MaxEnt score and next distal natural 3′ ss score. Non-filled circles represent exons with DMD/IMD-associated mutations, while grey-filled circles represent exons with BMD-associated mutations. Black bordered circles indicate exons without PESE disruptions. Blue bordered circles indicate exons with PESE disruption. Exon numbers are indicated beside circles.

In line with previous reports [19]–[22], our data indicate that SRE disruption/creation is not the only factor determining exon-skipping, since 6 out of 11 mutations disrupting PESE octamers in in-frame exons were found in IMD/DMD patients (Table S1). In a recent work, Flanigan et al. 2011 reported that exon-skipping occurs in a subset of exons, proposing a model in which a weak exon definition context, defined by a weak 3′ ss and low ESE density, is necessary for mutation-associated exon-skipping [23]. In our cohort, we identified BMD nonsense/frameshift mutations in exons 9, 28, 37, 71 and 74. To our knowledge, this is the first report of exon-skipping events in exons 9 and 28. According to the model of Flanigan et al, exons 37 and 71 present weak 3′ ss and a low ESE density, while exon 9 exhibits the lowest ESE density in our subset of exons (Figure 4). Exons 28 and 74, however, present high ESE densities and strong acceptor splice sites. Furthermore, in exons 3 and 33, presenting a weak exon definition context similar to other skipped exons, PESE disruption does not induce exon-skipping as cDNA analysis or the patient's phenotype indicate. Our data suggest that factors other than a weak 3′ ss and low ESE density may also influence the skipping capability of exons. The genomic context probably displays a relevant role in many exons. Mutations c.3850G>T (exon 28) and c.3982C>T (exon 29) induce both double skipping of exon 28 and 29, while mutation c.3786+1G>A (exon 27) produces significant amounts of transcripts showing skipping of exons 27 to 29. Other splice site mutations, such as c.1332-9A>G (exon 12) and c.10086+5G>C (exon 69), showed transcripts presenting skipping of the mutated exon and other neighbouring exons. These findings suggest a kind of priming effect in some exons, as previously observed in other genes [50], and reinforce the idea that the overall pre-mRNA architecture might be involved in the splicing process [51].

We observed that most splice site mutations induce variable levels of multiple alternatively spliced transcripts. Probably for this reason, splice site mutations are more frequent in BMD than in DMD. The splicing pathway differs substantially from one mutation to another, with main outcomes consisting in exon-skipping or activation of alternative sites. Intron retention was found only in one case and involved the smallest *DMD* intron (107 bp), in line with previous findings [52]. Several mutations induce more than one pathway at the same time. For this reason, predicting how these mutations will affect splicing patterns without mRNA studies is challenging. Algorithms such as HSF and MaxEnt are useful tools to predict abrogation or reduction of splice site function, creation of new sites and presence of cryptic sites. Changes located in non-canonical AG or GT nucleotides, slightly reducing the site strength are expected to induce significant amounts of normally spliced transcripts. However, this can not be generalized to all mutations. While mutations c.3603+2dupT and c.9563+5G>C induced significant amounts of normally spliced transcripts, c.3432+3A>T and c.10086+5G>C induced mainly aberrant transcripts (Table 2). These findings and the variety of observed splicing outcomes indicate that factors other than splice sites influence the final pattern. Multiple factors have been suggested to determine the splicing pathway, including the sequence context of the affected splice site, exon and intron length, RNA secondary structures, and conservation of the reading frame [51], [53], [54]. The abundance of cryptic splice sites has been suggested as a main factor determining whether a mutation induces exon-skipping or cryptic splice site activation [55]. Confirming a previous work [56], our data indicate that the availability of cryptic splice site does not determine the main splicing pathway, since numerous potential sites are found in most analyzed exons according to HSF and MaxEnt predictions. Habara et al. proposed that in +1G>A mutations a strong exon recognition, resulting from the combination of a high 3′ ss score and a long exon length, is necessary for cryptic site activation [56]. Our data indicate that the splicing pathway in 5′ ss mutations is determined by the interplay between the relative strength of 3′ ss and the density of ESE elements (Figure 3). Cryptic site activation occurs in those exons that present a strong 3′ ss compared with the next distal exon, while weak 3′ ss lead to exon-skipping. However, two exceptions are found in our subset of exons. Exon 26 presenting a weak 3′ ss showed cryptic site activation, while exon 69 presenting a moderately strong 3′ ss showed exon-skipping (Figure 3C). We hypothesize that a high density of ESE motifs may compensate a weak 3′ ss, leading to activation of alternative splice sites. In the other hand, a low ESE density in a moderately strong 3′ ss context may contribute to exon-skipping.

Precise identification of *DMD* mutations and their consequences on mRNA and protein expression is essential to provide accurate genetic counseling in dystrophinopathy families and to include patients in mutation suppression therapies. Our results support and extend previous findings showing that 3′ ss strength and density of regulatory elements are determinant factors of the splicing pathway in mutations affecting splicing signals. However, other factors such as the genomic context may also play a relevant role, suggesting a more complex model. Understanding the splicing code and developmenting computational splicing models will be of great value to predict pathological effects of DNA variants in molecular diagnosis of dystrophinopathy and other diseases, and to design more efficient molecules for splicing modulation therapies.

## Supporting Information

Table S1 **Nonsense and frameshift mutations located in in-frame exons were analyzed using different matrices to predict creation or disruption of splicing regulatory elements.** For ESE matrices, 1 represents disruption of at least one ESE motif while 0 represents no disruption; for ESS matrices, 1 represents creation of an ESS motif while 0 represents no creation.
(DOCX)

Table S2 **Primer pairs used to perform semi-quantitative QF-PCR in muscle cDNA are listed.** Primer name indicates its exonic location in the cDNA. For each amplicon, length of normal transcript and analyzed mutations are indicated.
(DOC)
