# Supplementary material for: Interplay between DMD Point Mutations and Splicing Signals in Dystrophinopathy Phenotypes
Source: PLoS One. 2013 Mar 25;8(3):e59916. doi: 10.1371/journal.pone.0059916 (PMC3607557; doi:10.1371/journal.pone.0059916)
Supplement: Table S1 — Nonsense and frameshift mutations located in in-frame exons were analyzed using different matrices to predict creation or disruption of splicing regulatory elements. For ESE matrices, 1 represents disruption of at least one ESE motif while 0 represents no disruption; for ESS matrices, 1 represents creation of an ESS motif while 0 represents no creation. (DOCX) [file pone.0059916.s001.docx]

| **Phenotype** | **Mutation** | **Exon** | **ESE finder** | **Rescue-ESE** | **PESE** | **EIE** | **Tra2-β 9G8** | **Sironi’s ESS** | **Wang’s ESS** | **Fas-ESS** | **PESS** | **IIE** | **hnRNP A1** |
| --- | --- | --- | --- | --- | --- | --- | --- | --- | --- | --- | --- | --- | --- |
| DMD | c.114_115del | 3 | 0 | 1 | 1 | 1 | 1 | 0 | 0 | 0 | 0 | 0 | 0 |
| DMD | c.174_175del | 3 | 1 | 0 | 0 | 0 | 1 | 0 | 0 | 0 | 0 | 0 | 1 |
| BMD | c.883C>T | 9 | 0 | 0 | 1 | 0 | 0 | 0 | 0 | 0 | 0 | 1 | 0 |
| DMD | c.1510C>T | 13 | 0 | 1 | 1 | 1 | 1 | 0 | 0 | 0 | 0 | 0 | 1 |
| DMD | c.1510dup | 13 | 0 | 1 | 0 | 0 | 1 | 0 | 0 | 0 | 0 | 0 | 0 |
| DMD | c.1638G>A | 14 | 1 | 0 | 0 | 0 | 0 | 0 | 0 | 0 | 0 | 0 | 0 |
| DMD | c.3427C>T | 25 | 0 | 0 | 0 | 1 | 0 | 0 | 0 | 0 | 0 | 0 | 0 |
| DMD | c.3511G>T | 26 | 1 | 1 | 0 | 1 | 1 | 1 | 0 | 1 | 0 | 0 | 0 |
| DMD | c.3578T>A | 26 | 1 | 0 | 0 | 1 | 0 | 0 | 0 | 0 | 0 | 0 | 0 |
| BMD | c.3850G>T | 28 | 0 | 1 | 1 | 1 | 1 | 0 | 0 | 0 | 0 | 0 | 1 |
| IMD | c.3982C>T | 29 | 1 | 0 | 0 | 1 | 0 | 1 | 0 | 0 | 0 | 0 | 1 |
| DMD | c.4099C>T | 30 | 1 | 1 | 1 | 1 | 0 | 0 | 0 | 0 | 0 | 0 | 0 |
| DMD | c.4527T>G | 33 | 1 | 0 | 0 | 0 | 0 | 0 | 0 | 0 | 0 | 1 | 1 |
| DMD | c.4558G>T | 33 | 0 | 1 | 1 | 1 | 1 | 0 | 0 | 1 | 0 | 1 | 0 |
| DMD | c.4838G>A | 34 | 1 | 0 | 0 | 1 | 1 | 1 | 0 | 1 | 0 | 0 | 1 |
| DMD | c.5131C>T | 36 | 1 | 0 | 0 | 1 | 0 | 1 | 0 | 0 | 0 | 0 | 1 |
| DMD | c.5139_c.5140delinsT | 36 | 1 | 1 | 1 | 1 | 1 | 1 | 0 | 0 | 0 | 1 | 1 |
| BMD | c.5287C>T | 37 | 0 | 0 | 1 | 1 | 0 | 0 | 0 | 0 | 0 | 1 | 0 |
| DMD | c.5308A>T | 37 | 1 | 1 | 0 | 1 | 0 | 0 | 0 | 0 | 0 | 1 | 0 |
| IMD | c.5371C>T | 38 | 0 | 0 | 0 | 1 | 0 | 0 | 0 | 0 | 1 | 0 | 0 |
| DMD | c.5530C>T | 39 | 0 | 1 | 0 | 1 | 1 | 1 | 0 | 1 | 0 | 0 | 0 |
| DMD | c.5611A>T | 40 | 0 | 1 | 0 | 1 | 1 | 0 | 0 | 0 | 0 | 0 | 0 |
| DMD | c.5613del | 40 | 1 | 0 | 0 | 0 | 1 | 0 | 0 | 0 | 0 | 0 | 0 |
| DMD | c.5646C>A | 40 | 1 | 1 | 0 | 1 | 0 | 0 | 0 | 0 | 0 | 0 | 0 |
| DMD | c.6973C>T | 48 | 0 | 0 | 0 | 0 | 0 | 0 | 0 | 1 | 0 | 1 | 1 |
| DMD | c.8944C>T | 60 | 0 | 1 | 0 | 1 | 0 | 0 | 0 | 0 | 0 | 1 | 0 |
| DMD | c.8955dup | 60 | 1 | 0 | 0 | 1 | 0 | 0 | 0 | 0 | 0 | 1 | 0 |
| DMD | c.9337C>T | 64 | 1 | 0 | 0 | 0 | 0 | 0 | 0 | 0 | 0 | 0 | 0 |
| DMD | c.9348dup | 64 | 1 | 1 | 1 | 1 | 1 | 1 | 0 | 0 | 0 | 0 | 1 |
| BMD | c.10231_10235del | 71 | 1 | 0 | 1 | 1 | 0 | 0 | 0 | 0 | 0 | 1 | 0 |
| BMD | c.10235del | 71 | 0 | 0 | 1 | 0 | 0 | 0 | 0 | 0 | 0 | 1 | 0 |
| BMD | c.10409dup | 74 | 0 | 0 | 0 | 0 | 0 | 0 | 0 | 1 | 1 | 1 | 1 |
|  | Fisher Exact Test *P* value |  | 0,0755 | 0,1959 | 0,0112 | 0,3461 | 0,3606 | 0,2964 | 1 | 1 | 0,3448 | 0,0185 | 1 |
