# Supplementary material for: Interplay between DMD Point Mutations and Splicing Signals in Dystrophinopathy Phenotypes
Source: PLoS One. 2013 Mar 25;8(3):e59916. doi: 10.1371/journal.pone.0059916 (PMC3607557; doi:10.1371/journal.pone.0059916)
Supplement: Table S2 — Primer pairs used to perform semi-quantitative QF-PCR in muscle cDNA are listed. Primer name indicates its exonic location in the cDNA. For each amplicon, length of normal transcript and analyzed mutations are indicated. (DOC) [file pone.0059916.s002.doc]

| **Forward primer** | **Reverse primer** | **Length (bp)** | **Analyzed mutations** |
| --- | --- | --- | --- |
| 5'UTRF: AAGCTGCTGAAGTTTGTTGGTTTCTCA | 8R: GCTCACTTGTTGAGGCAAAACTTGGA | 844 | c.94-1G>T, c.265-1G>A |
| 9F: CGATTCAAGAGCTATGCCTAC | 15R: CAGTTGTGTGAATCTTGTTCACTGC | 883 | c.1332-9A>G, c.1704+2T>A |
| 17F: CATGCTCAAGAGGAACTTCC | 22R: CTCCTGATAGCGCATTGGTGG | 685 | c.2803+1del |
| 24F: GCAGCTGAAACAGTGCAGACT | 28R: CTAGCACCTCAGAGATTTCCTC | 661 | c.3432+3A>T |
| 25F: CAATTCAGCCCAGTCTAAAC | 30R: AGCTGCGTCCACCTTGTCTGC | 914 | c.3603+1G>A, c.3603+2dupT, c.3786+1G>A, c.3850G>T, c.3982C>T |
| 35F: TTGGGCAAGAAGGAGACGTTGG | 38R: TCAGCCTCCAGTGGTTCAAGCAA | 476 | c.5287C>T |
| 35F: TTGGGCAAGAAGGAGACGTTGG | 40R: CCTTTCATCTCTGGGCTCAG | 807 | c.5444A>G |
| 43F: GCTACAGGAAGCTCTCTCC | 48R: TTCAAGCTGCCCAAGGTCTT | 763 | c.6913-1G>A |
| 59F: CCCGTGGGCGATCTCCTCATT | 66R: TGCTTTACACAGGGAAATGATGCCAG | 743 | c.9225-647A>G |
| 59F: CCCGTGGGCGATCTCCTCATT | 68R: CCAGTCTCATCCAGTCTAGG | 978 | c.9563+5G>C |
| 67F: GGTGAAGTTGCATCCTTTGG | 70R: GCCCATTCGGGGATGCTTCG | 434 | c.10086+5G>C |
| 67F: GGTGAAGTTGCATCCTTTGG | 73R: TTTCCATTTCTGCTAGCCTGC | 600 | c.10235del |

Table 3 suppemental
